# Evolution of the mitochondrial genome in snakes: Gene rearrangements and phylogenetic relationships

**DOI:** 10.1186/1471-2164-9-569

**Published:** 2008-11-28

**Authors:** Jie Yan, Hongdan Li, Kaiya Zhou

**Affiliations:** 1 Jiangsu Key Laboratory for Biodiversity and Biotechnology, College of Life Sciences, Nanjing Normal University, Nanjing 210046, PR China

## Abstract

**Background:**

Snakes as a major reptile group display a variety of morphological characteristics pertaining to their diverse behaviours. Despite abundant analyses of morphological characters, molecular studies using mitochondrial and nuclear genes are limited. As a result, the phylogeny of snakes remains controversial. Previous studies on mitochondrial genomes of snakes have demonstrated duplication of the control region and translocation of *trnL *to be two notable features of the alethinophidian (all serpents except blindsnakes and threadsnakes) mtDNAs. Our purpose is to further investigate the gene organizations, evolution of the snake mitochondrial genome, and phylogenetic relationships among several major snake families.

**Results:**

The mitochondrial genomes were sequenced for four taxa representing four different families, and each had a different gene arrangement. Comparative analyses with other snake mitochondrial genomes allowed us to summarize six types of mitochondrial gene arrangement in snakes. Phylogenetic reconstruction with commonly used methods of phylogenetic inference (BI, ML, MP, NJ) arrived at a similar topology, which was used to reconstruct the evolution of mitochondrial gene arrangements in snakes.

**Conclusion:**

The phylogenetic relationships among the major families of snakes are in accordance with the mitochondrial genomes in terms of gene arrangements. The gene arrangement in *Ramphotyphlops braminus *mtDNA is inferred to be ancestral for snakes. After the divergence of the early *Ramphotyphlops *lineage, three types of rearrangements occurred. These changes involve translocations within the *IQM *tRNA gene cluster and the duplication of the CR. All phylogenetic methods support the placement of *Enhydris plumbea *outside of the (Colubridae + Elapidae) cluster, providing mitochondrial genomic evidence for the familial rank of Homalopsidae.

## Background

Snakes are a large group of reptiles with a broad range of morphological features, of which many are evolutionarily selected by their habitats. Snakes have conventionally been divided into two groups. The fossorial scolecophidians (blindsnakes and threadsnakes) are small snakes with a small gape size that feed on small prey on a frequent basis. The second major group, the alethinophidians (or "true snakes") are more ecologically diverse and most species feed on relatively large prey on an infrequent basis. True snakes are further split into the Henophidia and the Caenophidia. The caenophidians, which are also called advanced snakes, include the aquatic genus *Acrochordus *and the Colubroidea. The Colubroidea is subdivided into the families Atractaspididae, Elapidae, Viperidae, and Colubridae. A small colubrid subfamily, Homalopsinae, was first attributed familial rank by Günther in 1864[1], and was recognized as subfamily in the 20^th ^century by most researchers until it was reassigned familial status in recent years [2-4].

Recent phylogenetic analyses, based primarily on molecular analyses of a few mitochondrial or nuclear genes failed to reach a consensus in several aspects [2,3,5,6]. For instance, the composition of the family Colubridae, the putative paraphyly and the hierarchical structuring into subfamilies remain contentious issues. The mitochondrial genome has several advantages for phylogenetic studies [7,8], and has been widely used in constructing animal phylogeny including snakes [9].

Previous studies of snake mitochondrial genomes have demonstrated that duplication of the control region and translocation of *trnL *are two visible features of the alethinophidian mtDNAs [9,10]. Moreover, translocation and pseudogenization of *trnP *have been found in some caenophidian snakes [10]. The Texas threadsnake (*Leptotyphlops dulcis*) possesses a different gene arrangement and loses its origin of light strand replication (O_L_) [11]. In the present study, we determined complete mitochondrial DNA sequences from four snake families. The sequence information allowed the gene organizations, mitochondrial genome evolution, and phylogenetic relationships among these major snake families to be identified.

## Results

### Characteristics of the snake mitochondrial genomes

The general characteristics of four snake (Table 1) mitochondrial genomes are summarized in Table 2. These complete mt genomes range from 16,397 to 17,548 bps in size. Length differences are largely due to the variation in lengths and/or numbers of the control region. In three of the four genomes (*Deinagkistrodon acutus*, *Naja naja*, and *Enhydris plumbea*), two control regions are found in the positions identical to those in other alethinophidian taxa mtDNAs. MtDNA sequence for *Ramphotyphlops braminus *is considerably smaller due to the absence of the control region duplication. All the genomes contain 13 protein-coding genes, 2 rRNAs genes, and 22 tRNAs genes. The base compositions in these mtDNAs are skewed similarly to other vertebrate mtDNAs [12], with more A-T base pairs than G-C base pairs and greater A+C content in the gene-rich strand than in the gene-poor strand.

**Table 1:** List of taxa used in this study

**Family**	**Species**	**GenBank** **Accession no.**	**Reference**
Ingroup			
Scolecophidia			
Typhlopidae	*Ramphotyphlops braminus*	DQ343649	This study
Leptotyphlopidae	*Leptotyphlops dulcis*	AB079597	Kumazawa, 2004
Alethinophidia			
Henophidia			
Boidae	*Boa constrictor*	AB177354	Dong and Kumazawa, 2005
Pythonidae	*Python regius*	AB177878	Dong and Kumazawa, 2005
Cylindrophiidae	*Cylindrophis ruffus*	AB179619	Dong and Kumazawa, 2005
Xenopeltidae	*Xenopeltis unicolor*	AB179620	Dong and Kumazawa, 2005
Caenophidia			
Colubridae	*Dinodon semicarinatus*	AB008539	Kumazawa et al., 1998
	*Pantherophis slowinskii*	DQ523162	Jiang et al., 2007
Elapidae	*Naja naja*	DQ343648	This study
Homalopsidae	*Enhydris plumbea*	DQ343650	This study
Viperidae	*Deinagkistrodon acutus*	DQ343647	This study
	*Ovophis okinavensis*	AB175670	Dong and Kumazawa, 2005
	*Agkistrodon piscivorus*	DQ523161	Jiang et al., 2007
Acrochordidae	*Acrochordus granulatus*	AB177879	Dong and Kumazawa, 2005
Outgroup			
Amphisbaenidae	*Amphisbaena schmidti*	AY605475	Macey et al., 2004
Scincidae	*Eumeces egregius*	NC_000888	Kumazawa and Nishida, 1999
Iguanidae	*Iguana iguana*	AJ278511	Janke et al., 2001
Varanidae	*Varanus komodoensis*	AB080275/AB080276	Kumazawa and Endo, 2004

**Table 2:** General characteristics of four snake mitochondrial genomes

**Taxa**	**Genome Length (bp)**					**G + C nucleotide content (%)**				
	
	**Total**	**Protein coding**	**rRNAs**	**tRNAs**	**Control region**	**Total**	**Protein coding**	**rRNAs**	**tRNAs**	**Control region**
*Ramphotyphlops braminus*	16397	11286	2366	1456	1278	42.9	42.8	46.9	41.1	39.3
*Deinagkistrodon acutus*	17548	11287	2384	1430	2410	42.1	43.0	41.2	42.2	38.7
*Naja naja*	17213	11241	2426	1435	2057	41.7	41.7	42.5	43.5	39.8
*Enhydris plumbea*	17397	11261	2398	1416	2238	40.6	40.2	42.1	42.9	39.1

**Figure 1:**
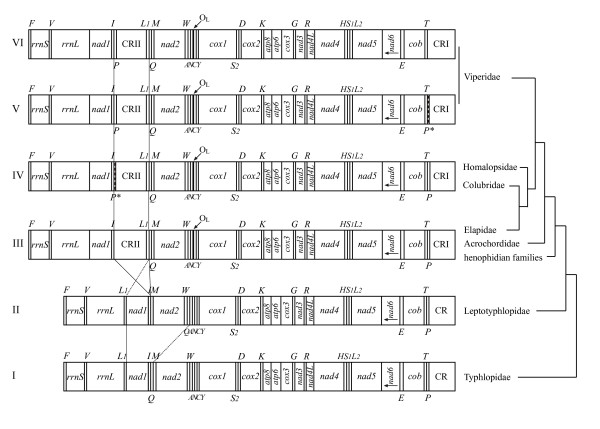
**Comparison of mitochondrial gene organizations of snakes**. Gene arrangements are presented for the following six types of snakes. I: *Ramphotyphlops braminus*; II:*Leptotyphlops dulcis*; III: *Naja naja*, *Acrochordus granulatus*, *Boa constrictor*, *Cylindrophis ruffus*, *Python regius*, *Xenopeltis unicolor*; IV: *Dinodon semicarinatus*, *Pantherophis slowinskii*, *Enhydris plumbea*; V: *Ovophis okinavensis*; VI: *Deinagkistrodon acutus, Agkistrodon piscivorus*. *L*_1_, *L*_2_, *S*_1_, *S*_2_. P* represent genes for *trnL(UUR)*, *trnL(CUN)*, *trnS(AGY)*, *trnS(UCN)*, and a pseudogene for *trnP*. tRNAs transcribed from the gene-rich and gene-poor strand were specified by noting their names above and below the gene map respectively. The tree topology on the right side was simplified from Fig. 2.

By comparing the gene arrangements in 14 known snake mitochondrial genomes, six types of gene organization were summarized and shown in Figure 1. Type I and II represent gene organizations of two scoleophidian snakes. They both have similar organization to that of typical vertebrate except the absence of an identifiable origin of light strand replication (O_L_) in the scolecophidian mtDNAs. Moreover, in the mitochondrial genomeof *Leptotyphlops dulcis *(Type II), *trnQ *was translocated from the *IQM *tRNA gene cluster to the *WANCY*cluster (the tRNA genes are abbreviated by single letters representing the amino acids to be decoded, and the sense strand of the underlined tRNA genes is the heavy strand), giving rise to the *IM *plus *WQANCY*organizations [11]. The common features of the remaining four arrangements (type III to VI) are the duplication of the control region and translocation of *trnL*, which have been noted in previous studies [9,10]. Six alethinophidian snakes from six families shared type III, and type IV represented an arrangement shared between Colubridae and Homalopsidae. In these two types, the functional *trnP *is located next to CRI (location in typical vertebrate mtDNA), with or without a pseudogene (P*) close to CR II (there are two control regions in most snake mitogenomes except scolecophidian snakes shown as type I and II). In several previous studies, the viperid snakes appear to have another gene rearrangement in which a functional *trnP *moved from the end of CR I to that of CR II [9,10,13]. In this study, the positional switch of *trnP *is found for another viperid taxon *Deinagkistrodon acutus*, and is not found for the nonviperid snakes (Fig. 1). Gene arrangements in viperids can be summarize as type V and VI, with only a difference in the pseudogene (P*).

### Phylogenetic analyses

**Figure 2:**
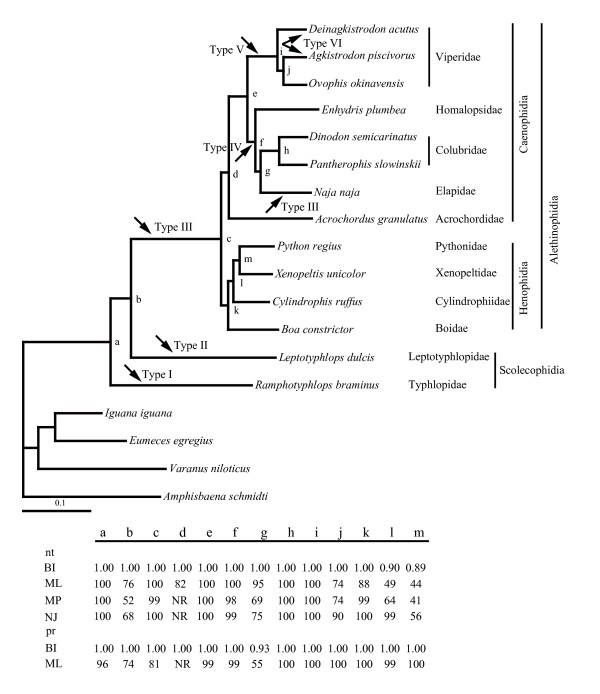
**Phylogenetic analyses of the amino acid and nucleotide sequence data sets**. The phylogram shown is the best maximum likelihood tree (-lnL = 34008.56) obtained from the nucleotide data set. Bar represents 0.1 mutations per site. Nodes receiving support by one or more of the applied phylogenetic methods, *i. e.*, Bayesian inference (BI), Maximum likelihood (ML), maximum parsimony (MP), Neighbor-joining (NJ) are labelled with lowercase letters. nt = values obtained from the nucleotide data set; and pr = values obtained from the amino acid data set. Bootstrap and Bayesian inference values are listed in the table. NR means that the corresponding nodal relationship was not reconstructed in the tree topology. Types I to VI are corresponding to those in Fig. 1.

The results of the phylogenetic reconstructions are displayed in Figure 2. Our phylogenetic estimates strongly support *Ramphotyphlops braminus *being the sister lineage to all remaining species sampled, and that the scolecophidian snakes do not form a monophyletic grouping. All of the analyses were congruent in receiving the monophyly of Alethinophidia (c in Figure 2). Within this monophyletic clade, Henophidia and Caenophidia were clustered as monophyletic sister groups (k and d, respectively). Our estimates of relationships among the henophidians are similar to that of Dong and Kumazawa [9] in the rejection of the traditional expectations of a sister-group relationship between boids and pythonids (m in Fig. 2) and the deep divergence of non-macrostomatan cylindrophiids (l in Fig 2). Consistent with recent molecular phylogenies of caenophidian snakes [2,3,5], we find support for the Viperidae as the deepest diverging lineage within the Colubroidea, sister to a clade containing colubrids, elapids, and homalopsids. The position of *Enhydris plumbea *outside of the (Colubridae + Elapidae) cluster seems strongly supported by all phylogenetic methods (f in Fig. 2). Traditional placement of *Enhydris plumbea *within Colubridae based on morphology was not retrieved by our analyses. Node d, corresponding to the position of *Acrochordus granulatus*, is the only one not reconstructed in all tree-building methods. In Bayesian and ML tree based on nucleotide data set and Bayesian tree on amino acid data set, the placement of Acrochordidae to the sister group to Colubroidea was well supported.

To further substantiate the above premises, we performed the SH statistical test [14] on both amino acid and nucleotide data sets that allows comparison of alternative phylogenetic hypotheses. Results of the SH test strongly rejected the placement of *Enhydris plumbea *within the colubrids cluster (*P *< 0.001). The monophyly of scolecophidian snakes was not rejected using both amino acid and nucleotide acid data sets (P > 0.05).

## Discussion

### Evolution of snake mitochondrial genomes

Based on the phylogenetic relationships among the tested snakes and the comparisons of their gene organizations (Fig. 1, 2), we estimated the processes of evolutionary events occurred in snake mitochondrial genomes. In early snake lineages (type I and II), gene arrangements are similar to that of typical vertebrate, but O_L _was lost within the *WANCY*tRNA gene cluster. Incompatible with the commonly accepted view on monophyly of scolecophidian snakes [15-18], our phylogenetic estimates strongly supports *Ramphotyphlops braminus *being the sister lineage to all remaining species sampled. Given that the monophyly of scolecophidian snakes was not rejected in SH statistical test, loss of O_L _may occur in two different scenarios, independently (if nonmonophyly) or descend from a common ancestry (if monophyly). After the divergence of the *Ramphotyphlops *lineage, changes involving the *IQM *cluster took place. First in type II, *trnQ *underwent a long distance translocation (~1.2 kb) from one gene cluster to another (Fig. 1, 2). Subsequently, in the early alethinophidian lineage, the control region was duplicated and *trnL *relocated to the *IQM *cluster, giving rise to type III which is present in most alethinophidian snakes (including henophidians, *Acrochordus *and *Naja*). New types emerged during the split in Caenophidia. Type IV is found in two branches, *Dinodon semicarinatus*, *Pantherophis slowinskii *(Colubridae), and *Enhydris plumbea *(Homalopsidae), and characteristic changes (P*) likely appeared ahead of node f, which then disappeared in Elapidae. It is also conceivable that the present of P* was resulted from independent evolution in Colubridae and Homalopsidae. Distinct arrangements (type V and VI) were found in viperids, suggesting that *trnP *was translocated in early stage of the viperid radiations [9]. Type VI, with no pseudogenes close to CRI, was found in two paraphyletic taxa, suggesting that P* could have been independently eliminated.

### Familial rank of Homalopsidae

The Homalopsinae have been generally recognized as a valid monophyletic clade within the Colubridae [19,20] and assigned a subfamilial rank, despite they being assigned familial [1] or tribal [21] status historically. Recent molecular studies placed the Homalopsinae as the sister group to most other members of the Colubroidea [2,3,5], and a familial status has been reassigned accordingly [2-4].

In this study, the placement of *Enhydris plumbea*, a representative of the Homalopsidae, as the sister lineage to the Colubridae + Elapidae clade was strongly supported by all phylogenetic methods. Moreover, SH test strongly rejected the hypothesis that *Enhydris plumbea *falls within the colubrids cluster (*P *< 0.001). The familial rank of Homalopsidae is therefore considered well-supported. Our work for the first time establishes the monophyly and distinctiveness of this family with phylogenetic evidence derived from complete mitochondrial genome sequences.

## Conclusion

In this study, six types of mitochondrial gene arrangement in snakes are summarized. Two notable features of the alethinophidian mtDNA, duplication of the CR and translocation of *trnL*, are presented. The gene arrangement in *Ramphotyphlops braminus *mtDNA is indentical to that found in typical vertebrates, suggesting an ancestral arrangement. The well supported phylogenetic topology helps to reconstruct the evolution of mitochondrial gene arrangements in snakes. We propose that, after the divergence of the early *Ramphotyphlops *lineage, three types of changes involving the *IQM *gene cluster occurred. These include the translocation of *trnQ *in the early *Leptotyphlops *lineage, the duplication of CR and translocation of *trnL *in the early alethinophidian lineage, and the translocation of *trnP *in the early viperid lineage. All phylogenetic methods support the placement of *Enhydris plumbea *outside of the (Colubridae + Elapidae) cluster, providing mitochondrial genomic evidence for the familial rank of Homalopsidae. The monophyly of Scolecophidia is not rejected in our study. However, a more comprehensive sampling of snake mitochondrial genomes is necessary to further refine the phylogenetic relationships among major groups of snakes.

## Methods

### Samples, DNA amplification, and sequencing

Snakes from three alethinophidian families and one scoleophidian family were sampled (Table 1). Total DNA was extracted from a small quantity (20 mg) of tissues by DNeasy Tissue Kit (Qiagen). Several short mtDNA fragments were amplified using Ex-*Taq *DNA polymerase (Takara) and sequenced in order to design taxon-specific primers. PCRs were performed in a MJ PTC-200 thermal cycler under the profile: 5 min at 95°C followed by 35 cycles of 95°C for 30 s, 50–55°C for 30 s, and 72°C for 90 s. PCR products of 1~2.5 kb were purified and then sequenced employing an ABI 310 or 3700 system with bi-directional and several internal primers. Short fragments were assembled into a continuous sequence. In the mtDNA sequences thus obtained, 37 individual genes were identified based on corresponding homologues from other vertebrates. Identification of tRNA genes was based on their secondary structures using software DNASIS 2.5 (Hitachi Engineering, Tokyo, Japan), whereas boundaries of rRNA genes and control regions were tentatively defined by the boundaries of adjacent coding genes. The mtDNA sequences, with annotations, have been deposited at GenBank (DQ343647–DQ343650).

### Taxa, alignment and phylogenetic analyses

We assembled 14 serpent ingroups with complete mitochondrial genomes available, and chose 4 taxa from 4 saurian families (*Amphisbaena schmidti *[22]; *Eumeces egregius *[23];*Iguana iguana *[24]; and *Varanus komodoensis *[25]) as outgroups (Table 1). Two data sets were prepared for concatenated amino acid sequences and for concatenated light-strand nucleotide sequences of the 12 protein genes. *Nad6*, the only protein gene encoded by the light strand, has been excluded for increased proportion of T and G in all codon positions due to the strand-specific base composition bias of mtDNAs. Multiple alignments were analyzed with the Gblocks program [26] to select conserved amino acid residues, which was later used as a backbone to align the corresponding nucleotide sequences.

The level of saturation in the whole codons, and at the first, second, and the third codon positions was independently analyzed using scatter plot graphics, by comparing the uncorrected p-distance with the distance calculated by applying the best-fit evolutionary model (GTR + I + G) selected by the Modeltest 3.7 [27]. The third positions of the protein genes were removed from the nucleotide data set because of high substitutional rates and consequent saturation as a source of noise in phylogenetic analyses. Thus a final alignment of 6566 bases was obtained.

Phylogenetic analyses were carried out using maximum likelihood (ML), Bayesian (BI), maximum parsimony (MP) and neighbor-joining (NJ) methods. The ML analyses with the nucleotide data set were conducted with PAUP*4.0b10 by a heuristic search with TBR branch swapping with 10 random taxon additions. The general reversible model (GTR + I + G) and parameters optimized by Modeltest 3.7 were used. Bayesian phylogenetic analyses of the nucleotide sequences were performed with MrBayes 3.1 [28] using a GTR + I + G model. The Markov chain Monte Carlo process was set to run four chains simultaneously. Posterior probabilities were calculated from the majority-rule consensus trees constructed after excluding the burn-in.

ML analyses with the amino acid data set were conducted using PUZZLE 5.2 [29] with the mtREV24 substitution matrix and amino acid frequency estimated from the data set. The Bayesian analyses of the amino acid data were conducted with MrBayes 3.0 using the mtREV24 + I + G model and an empirical amino acid frequency. The Bayesian tree and posterior probability values were obtained using the same procedures described above.

## Authors' contributions

JY conducted the amplification and sequence assembly of the mitochondrial genomes of three snakes. She collected, analyzed and summarized the data, and drafted the manuscript. HDL conducted the amplification and sequence assembly of the mitochondrial genomes of *Enhydris plumbea*. KYZ conceived the study and participated in its design and data interpretation, and preparation of the manuscript. All authors read and approved the final manuscript.
